# Global self-esteem and coping with stress by Polish students during the COVID-19 pandemic

**DOI:** 10.3389/fpubh.2024.1419771

**Published:** 2024-10-14

**Authors:** Ewa Kupcewicz, Anna Maria Cybulska, Daria Schneider-Matyka, Paweł Jastrzębski, Aleksandra Bentkowska, Elżbieta Grochans

**Affiliations:** ^1^Department of Nursing, Collegium Medicum, University of Warmia and Mazury in Olsztyn, Olsztyn, Poland; ^2^Department of Nursing, Pomeranian Medical University in Szczecin, Szczecin, Poland; ^3^Department of Emergency Medicine, Collegium Medicum, University of Warmia and Mazury in Olsztyn, Olsztyn, Poland; ^4^Hospital Emergency Department, Provincial Specialist Hospital in Olsztyn, Olsztyn, Poland

**Keywords:** challenges, coping, self-esteem, medical education, strategies, stress, university students

## Abstract

**Objectives:**

Students experience considerable stress and anxiety during the course of their studies, which has a significant impact on their health and hinders the learning process. There are many stressors that can intensify stress, which is why choosing the right strategies for coping with stress and self-esteem is so important.

**Methods:**

The study was conducted on 798 students of the School of Public Health at the University of Warmia and Mazury in majors: nursing, midwifery, emergency medicine and dietetics (subgroup 1; *n* = 428; 53.77%) and at the Faculty of Veterinary Medicine, major: veterinary medicine (subgroup 2; *n* = 368; 46.23%). The study employed the diagnostic survey method using a questionnaire technique including Rosenberg’s Self-Esteem Scale, MINI-COPE, PSS-10, and a self-questionnaire.

**Results:**

The scores obtained by over half of the students were indicative of low global self-esteem, whereas over 80% of the students felt stress at a high intensity. Moreover, it was shown that the veterinary medicine students demonstrated a higher intensity of coping strategies, i.e., active coping (*p* < 0.04) and planning (*p* < 0.02), than medicine students.

**Conclusion:**

The study revealed that students, irrespective of the major, experienced high levels of stress. Self-esteem had a significant impact on the stress level and methods of coping with difficult situations in students of medicine. A majority of the students applied positive styles of coping with stress.

## 1 Introduction

When beginning their studies, university students face the significant challenge of having to meet the requirements associated with entering the university environment. Moreover, young people encounter new stressors arising from the burdens of academic circles, interactions with teachers, busy schedules, and the need to transition from puberty to adulthood. All these challenges may expose students to more intensive stress and, as a consequence, make them more susceptible to health issues. Moreover, students may be unprepared to face additional stressors associated with family, social, academic and financial burdens, typical of this population (1).

Many scientists have observed that students of various majors feel a high level of stress, but it is the medical majors that are regarded as the most demanding and stressful studies, in which students have to acquire professional knowledge, skills and competences to ensure a high quality of patient care in their future practice. As part of their education, medical students face numerous challenges in clinical practice. They are exposed to academic pressure that is associated with clinical practice. The former is associated with a high emotional burden caused by frequent exams and the feeling of uncertainty, by an upset balance between personal life and studies (2). The latter includes a lack of skills and clinical experience, difficulties coping with patient death, intricacies of communication with patients, fear of making a mistake, handling emergencies in clinical conditions, and overcoming difficulties associated with gaps between theory and practice (3, 4).

Self-esteem is an emotional response to the difference between the “*real I*” and the “*ideal I*,” which can include only one dimension (values or competences), both dimensions (values and competences) or multiple dimensions of the academic, family or physical sphere (5, 6). According to Rosenberg’s opinion, self-esteem is a positive or negative attitude toward oneself, a type of global self-esteem. High self-esteem is a positive and favourable opinion of oneself as a person, while low self-esteem is expressed as a negative impression of oneself and a kind of self-rejection (7). Therefore, self-esteem is a complex and multi-faceted concept, which is often defined as self-assessment (8). High self-esteem is associated with higher motivation and solving problems more quickly, while lower self-esteem may cause mental disorders (9, 10), as well as an unfavourable image of oneself, including a loss of self-respect (11–13).

According to the findings of some studies, nursing students have high self-esteem (14–16), whereas according to others—it is moderate or low (17, 18). The self-esteem level is diverse even among professionally active nurses, and it is usually moderate (19–21). Moreover, some researchers report that self-esteem among nursing students can decrease considerably during nursing education (22). A literature review shows that there are many factors affecting students’ self-esteem (23). Self-esteem can also have an impact on professionalism (24), well-being and mental health (25), patient care quality (26), improving professional qualifications (27, 28), professional identity of nursing graduates and their motivation (29). According to some studies, there is a correlation between self-esteem and academic performance (30, 31).

Self-esteem is an important element required for an individual’s professional development and mental well-being. Self-esteem is of great importance in education as it affects professional behaviour and students’ mental well-being (32). Individuals with higher self-esteem have a better chance of a positive attitude toward their education, as well as their future professional career. Moreover, they are better prepared to cope with the stress and challenges of studying. Low self-esteem has a negative impact on academic performance, general well-being and professional choices (33).

There have been many studies that report the significant importance of the feeling of self-esteem as a stress moderator and emphasise its links with students’ mental well-being (34). According to multiple studies, students experience academic stress at various levels, and those stressed at a higher level demonstrate lower self-esteem (16, 34–38). Measuring the level of self-esteem among nursing students is important for several reasons. First, it can help identify students who may be at risk for academic difficulties or mental health problems. Second, it can help develop interventions to promote positive self-esteem and well-being among nursing students. Third, it can contribute to the overall improvement of nursing education programs and the quality of care provided by future nurses. Fourth, it can help identify pressure and protective factors that affect the self-esteem of nursing students. Further investigation is warranted to delve deeper into self-esteem issues. Therefore, the aim of this study was to examine the role of global self-esteem in coping with stress among Polish students and to seek predictors for stress intensity among the students under study during the COVID-19 pandemic.

## 2 Materials and methods

### 2.1 Settings and design

A group of 798 students of the School of Public Health at the University of Warmia and Mazury in Olsztyn in majors: nursing, midwifery, emergency medicine and dietetics (subgroup 1; *n* = 428; 53.77%) and at the Faculty of Veterinary Medicine majoring in veterinary medicine (subgroup 2; *n* = 368; 46.23%) were invited to participate in the study. The study was conducted between January and March 2022. The following were the inclusion criteria: age ≤ 30 years, signing an informed consent for participation in the study and completing the questionnaires. Students who failed to give such consent were excluded from the study. Following approval from the dean, the trained personnel distributed the paper questionnaires among individual study majors. The survey was conducted in direct contact with students in groups of about a dozen students. The participants were informed about the study objective and its anonymity, and they had an opportunity to ask questions and get comprehensive answers. They could withdraw from the study at any moment without giving a reason. It took approximately 15 min to complete the questionnaire. Altogether, 850 questionnaires were distributed, and 798 (93.88%) were taken for the final analysis. This study is part of a larger research project, and it was approved by the Senate Scientific Research Ethics Committee at the Olsztyn University in Olsztyn (No. 3/2021) and conducted according to the Declaration of Helsinki.

### 2.2 Research instruments

The study was conducted using a diagnostic survey and a questionnaire. The following research tools were used to collect the empirical data:

- The questionnaire, containing questions about demographic data, i.e., age, year of studies, gender, place of residence, extent of social contact restriction, number of hours of working from home, frequency of meals per day, preferred form of physical exercise and the extent of its limitations:
- The scale of global self-esteem SES *by Moriss Rosenberg in the Polish adaptation by Łagun M.* et al. (39);
- The PSS-10 questionnaire *by S. Cohen, T. Kamarck and R. Mermeldtein in the Polish version by Juczyński Z., Ogińska-Bulik N* (40);
- Coping with Stress Inventory—Mini-COPE *by Charles S. Carver in the Polish version by Juczyński Z., Ogińska-Bulik N* (40).

#### 2.2.1 SES global self-assessment scale by M. Rosenberg

The Rosenberg SES Self-Assessment scale contains 10 statements that refer to the beliefs of the person under study, and they are diagnostic in nature. A participant states the extent to which he/she agrees with each question by giving a response on the Likert scale from 1 to 4. The following scores were assigned to the responses: 1—I definitely agree, 2—I agree, 3—I disagree, 4—I definitely disagree. According to the recommendations of the scale authors, the positive statements (1, 2, 4, 6, 7) have a reverse score value when the results are calculated, so a higher score should be granted for the responses expressing a higher self-assessment level. The overall level of self-esteem is the sum of all the points whose theoretical distribution ranges from 10 to 40. The higher the score, the higher the self-esteem. A raw score for global self-esteem, following its conversion to standardised units in the sten scale, can be interpreted in accordance with its properties. The scores between 1 and 4 sten are regarded as low, 5 and 6—as average, whereas those from 7 to 10 sten—as high. The SES scale has good psychometric properties, and the Cronbach alpha ranges from 0.81 to 0.83 (39).

#### 2.2.2 PSS-10 questionnaire

The PSS-10 scale of perceived stress was used to assess the intensity of stress associated with the student’s life situation. The scale contains 10 questions about various subjective feelings related to personal problems and events, behaviours and methods of coping over the past month. A respondent can choose one out of five options in the Likert scale for each question, stating to what extent he/she agrees with a statement. The following values were assigned to each response: 0 = never, 1 = hardly ever, 2 = sometimes, 3 = quite often, 4 = very often. According to the recommendations of the scale authors, the score in answers to positive questions, i.e., 4, 5, 7, 8, was changed before the overall index of perceived stress was calculated, according to the rule: 0 = 4; 1 = 3; 3 = 1; 4 = 0. The overall score is the sum of all points, with the theoretical distribution from 0 to 40. The higher the score, the higher the experienced stress level. A raw score of the stress intensity is converted to standardised units in the sten scale and is interpreted in accordance with its properties. The scores between 1 and 4 sten were regarded as low, 5 and 6—as average, whereas those from 7 to 10 sten as high. In the original version, the scale of internal consistency, assessed on the basis of the Cronbach alpha, ranges from 0.84 to 0.86 for the three samples examined by Cohen et al. (40).

#### 2.2.3 Coping with stress inventory—Mini-COPE

The Mini-COPE inventory is a tool used for the measurement of coping, i.e., an assessment of the typical methods of responding and feeling in situations when one experiences severe stress. It contains 28 statements comprising 14 strategies for coping with stress. There are two statements referring to each strategy. A participant can choose one of four answers to each statement, to which the following values are assigned: 0—I hardly ever do this, 1—I rarely do this, 2—I often do this, 3—I nearly always do this. The scores are analysed separately for each strategy, or they can be grouped according to the common features of the scale structure. Therefore, there are seven groups of coping with stress strategies: active coping (active coping, planning, positive revalidation), helplessness (use of psychoactive substances, cessation of actions, self-blaming), seeking support (seeking emotional support, seeking instrumental support), avoidance behaviour (taking care of something else, denial, discharge), turning to religion, acceptance and sense of humour. The psychometric indices of the original version of Mini-COPE are regarded as good (Cronbach *α* = 0.70) (40).

### 2.3 Statistical analysis

The statistical analysis was conducted with STATISTICA v.13.3 (TIBCO, Palo Alto, CA, USA). The variance of the global self-esteem, stress intensity and the strategies of coping with stress in the subgroups under study was assessed with the ANOVA (F) analysis of variance with the Brown-Forsythe homogeneity test. The Pearson correlation was taken to examine the significance of the strength of the relationship between the variables under analysis, whereas the interpretation of the strength of the relationship was based on Guilford’s classification (41). The quantitative treatment of relationships between multiple independent (explaining) variables and a dependent (explained) variable is shown with the multiple regression analysis (41). The level of significance of *p* < 0.5 was adopted.

## 3 Results

### 3.1 Participants

A total of 798 students of the University of Warmia and Mazury in Olsztyn participated in the study, including 684 women (85.93%) and 112 men (14.07%). The mean age of the participants was 20.74 years (SD = 1.70). Nearly half of them (*n* = 380; 47.74%) lived with their family or with someone close, and the others lived in dormitories (25.88%, *n* = 206) or on their own (26.38%, *n* = 210). Nearly all the students declared to be satisfied with their health status. They spent more than 5.81 (SD = 2.66) hours a day working on a computer on average. More than 70% (*n* = 571) of the respondents reported that they had 3–4 meals a day, but usually not at the same time every day. A high percentage (93.34%) of the respondents reported that they reduced their social contacts to a medium or considerable extent. The COVID-19 pandemic also reduced physical activity to various extents. The students usually walked or jogged (*n* = 466; 58.54%) or went cycling (*n* = 159; 19.97%).

### 3.2 Variance of the global self-esteem, stress intensity and coping with stress strategies in the subgroups under study

**Figure 1:**
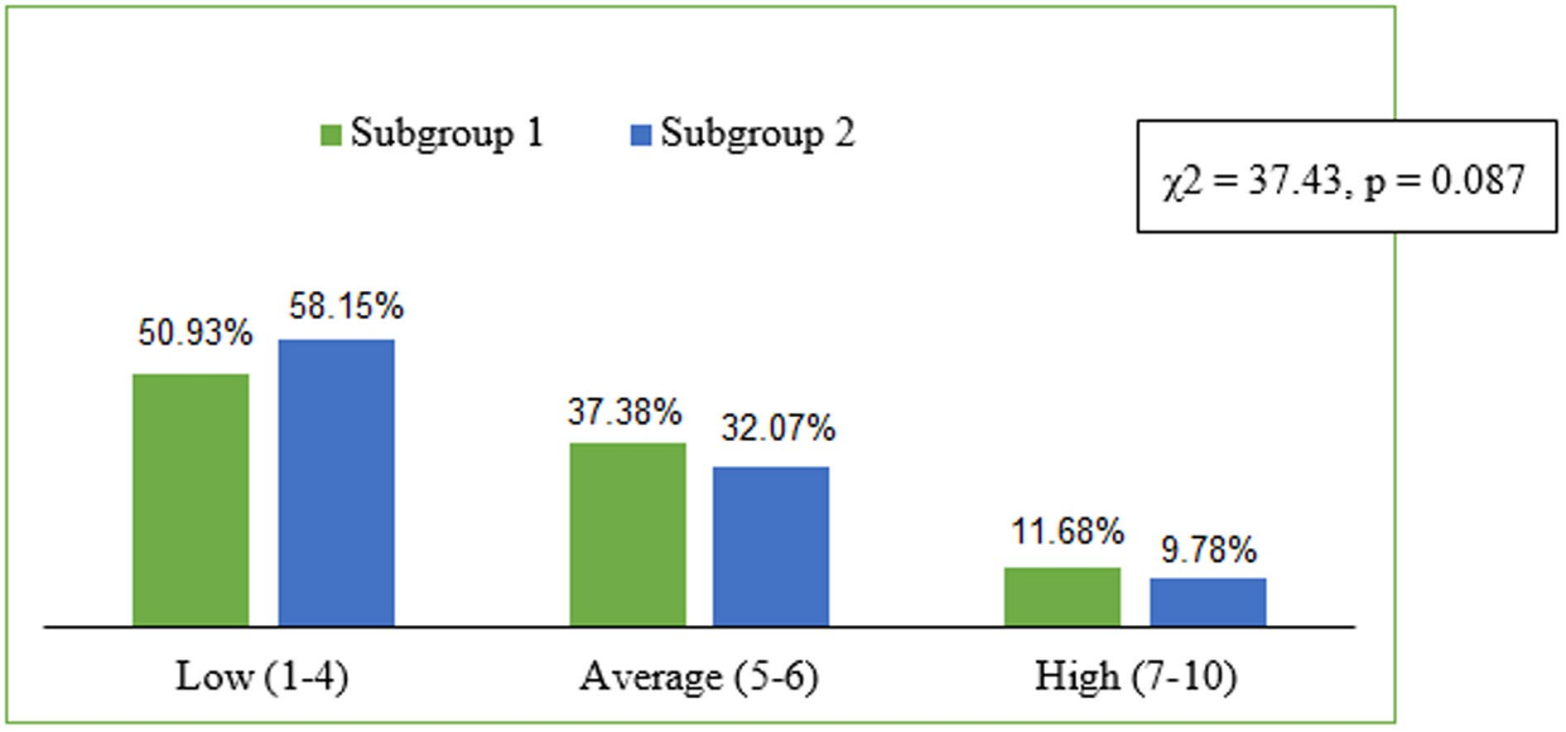
Distribution of self-esteem scores (SES) on the sten scale for the two subgroups.

The ANOVA (F) analysis of variance with the Brown-Forsythe homogeneity test revealed the differences in the global self-esteem between students in subgroups 1 and 2 (*F* = 7.07; *p* < 0.007). Students in subgroup 1 obtained a significantly higher overall global self-esteem index—27.68 points (SD = 4.53) on a scale between 10 and 40, compared with the students in subgroup 2 (*M* = 26.82; SD = 4.61; Table 1). Subsequently, the overall global self-esteem index was converted to standardised units and interpreted according to the properties characterising the sten scale. The analysis showed the distribution of the result structure on the sten scale for global self-esteem to be similar in both subgroups under study (*χ*^2^ = 37.43, *p* = 0.087). It was demonstrated that over half of the students had scores indicative of low self-esteem, *ca.* ⅓—scores on an average level and a small group—on a high level. The structure of the global self-esteem scores for the two groups on the sten scale is shown in Figure 1.

**Table 1:** Characteristics of the students.

Variables		Total *N* = 798	
		Number	%
Gender	woman	684	85.93
	man	112	14.07
Year of studies	first	337	42.34
	second	197	24.75
	third	262	32.91
Age (years) *M* = 20.74; *SD* = 1.70	<=20	386	48.49
	21	187	23.49
	> = 22	223	28.02
Place and form of residence during the COVID-19 pandemic	with family/someone close	380	47.74
	on their own	416	52.26
Number of hours spent working on a computer *M* = 5.81; *SD* = 2.66	<=3 h	169	21.23
	4 h-7 h	407	51.13
	> = 8 h	220	27.64
Number of meals *M* = 3.29; *SD* = 0.90	1–2	153	19.22
	3	335	42.09
	4	236	29.65
	5 and more	72	9.05
Reduction of physical exercise during the COVID-19 pandemic	no	265	33.29
	yes, to a small extent	155	19.47
	yes, to a medium extent	185	23.24
	yes, to a considerable extent	191	23.99
Subjective health status assessment during the COVID-19 pandemic	average	57	7.16
	good	568	71.36
	very good	171	21.48
Reduction of social contacts during COVID-19 pandemic	to a small extent	53	6.66
	to an average/medium extent	427	53.64
	to a considerable extent	255	32.04
	to a very large extent	61	7.66

**Figure 2:**
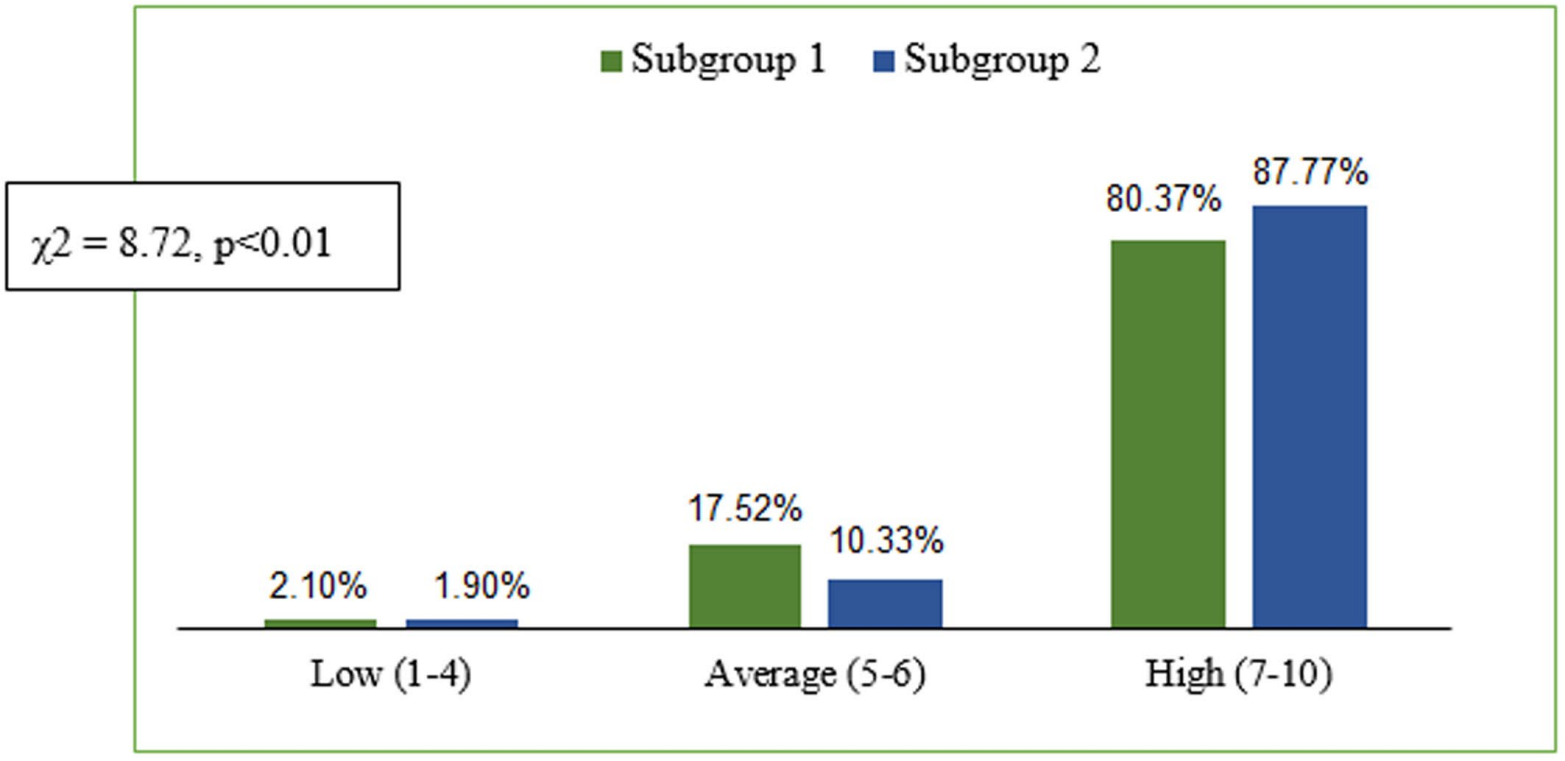
Distribution of stress intensity scores (PSS-10) for the two subgroups on the sten scale.

In subsequent analyses, significant differences were shown in the two samples in the level of stress intensity associated with the students’ situation over the previous month (*F* = 11.94; *p* < 0.0005). Students in subgroup 1 had lower average scores (*M* = 22.75; SD = 4.23) than those in subgroup 2 (*M* = 23.76; SD = 3.87). When the general stress intensity index was transformed to standardised units in the sten scale, it was found that a large group of students in both samples were exposed to a high level of stress, but the score distribution was significantly different than in the subgroups under study (*χ*^2^ = 8.72; *p* < 0.01). A significantly higher percentage of students in subgroup 2 (87.77%) than in subgroup 1 (80.37%) had scores within the range of 7–10 sten, indicative of high stress intensity. Students in subgroup 1 assessed their stress intensity as average significantly more often (17.52%) than those in subgroup 2 (10.33%). The data in Figure 2 show that an insignificant percentage of students in both subgroups had low scores, indicative of low stress intensity.

Further, a statistical analysis was performed of the coping with stress strategies used by the students under study. The seven-factor scale structure with 14 coping strategies was taken into consideration. The scores were strongly varied in the seven coping strategies. The group of strategies regarded as active coping strategies, manifesting themselves as taking actions aimed at improving the situation, students in subgroup 2 demonstrated a higher intensity of the coping strategies, i.e., active coping (*F* = 4.39; *p* < 0.04) and planning (*F* = 5.87; *p* < 0.02) than students in subgroup 1, while students in subgroup 1 chose the strategy of positive revalidation significantly more often (*F* = 13.81; *p* < 0.0002).

In a group of less effective strategies, referred to as helplessness, students in subgroup 1 obtained a significantly higher intensity of the cessation of action strategy (*F* = 10.54; *p* < 0.001) compared with students in subgroup 2. In stressful situations, students in subgroup 2 used a destructive strategy referred to as self-blaming more often (*F* = 35.23; *p* < 0.0001) than students in subgroup 1. The Denial strategy in the group referred to as avoidance behaviour was used significantly more often by students in subgroup 1 than in subgroup 2 (*F* = 16.09; *p* < 0.0001). It is noteworthy that the turning to religion strategy dominated in subgroup 1 (*F* = 7.93; *p* < 0.005). Praying and seeking consolation in meditation likely significantly increased the motivation of students in subgroup 1 rather than subgroup 2 to act in adverse stressful situations. Detailed data are provided in Table 2.

**Table 2:** Variance of the global self-esteem, stress intensity and coping with stress strategies in the subgroups under study *N* = 796.

Variables			Subgroup 1 *n* = 428 (53.77%)	Subgroup 2 *n* = 368 (46.23%)	ANOVA *(F)*	*p*-value
			M ± SD, Me, Min. – Max., 95% CI	M ± SD, Me, Min. – Max., 95% CI		
SES			27.68 ± 4.53, 28, 15–40, 27.25–28.12	26.82 ± 4.61, 27, 13–39, 26.35–27.29	7.07	*0.007*
PSS-10			22.75 ± 4.23, 23, 5–35, 22.36–23.16	23.76 ± 3.87, 24, 7–35, 23.36–24.16	11.94	*0.0005*
Mini-Cope	Active coping	Active coping	1.92 ± 0.64, 2, 0–3, 1.86–1.98	2.02 ± 0.69, 2, 0–3, 1.95–2.09	4.39	*0.04*
		Planning	1.92 ± 0.70; 2; 0–3; 1.85–1.99	2.04 ± 0.70, 2, 0–3, 1.97–2.11	5.87	*0.02*
		Positive revalidation	1.60 ± 0.73, 2, 0–3, 1.53–1.67	1.39 ± 0.82, 2, 0–3, 1.31–1.48	13.81	*0.0002*
	Helplessness	Use of psychoactive substances	0.71 ± 0.87, 1, 0–3, 0.62–0.79	0.60 ± 0.84, 0, 0–3, 0.52–0.69	2.98	0.08
		Cessation of actions	0.97 ± 0.76, 1, 0–3, 0.89–1.04	0.79 ± 0.75, 1, 0–3, 0.89–1.04	10.54	*0.001*
		Self-blaming	1.55 ± 0.87, 2, 0–3, 1.47–1.63	1.91 ± 0.83, 2, 0–3, 1.82–1.99	35.23	*0.0001*
	Seeking support	Seeking emotional support	1.98 ± 0.78, 2, 0–3, 1.91–2.06	1.97 ± 0.77, 2, 0–3, 1.89–2.05	0.02	0.88
		Seeking instrumental support	1.87 ± 0.78, 2, 0–3, 1.80–1.98	1.87 ± 0.78, 2, 0–3, 1.79–1.95	0.00	1.00
	Avoidance behaviours	Taking care of something else	1.81 ± 0.69, 2, 0–3, 1.74–1.87	1.80 ± 0.72, 2, 0–3, 1.73–1.87	0.03	0.87
		Denial	0.99 ± 0.77, 1, 0–3, 0.92–1.06	0.77 ± 0.75, 2, 0–3, 0.69–0.85	16.09	*0.0001*
		Discharge	1.67 ± 0.67, 2, 0–3, 1.61–1.73	1.72 ± 0.74, 2, 0–3, 1.64–1.79	0.83	0.36
	Turning to religion		0.88 ± 0.97, 1, 0–3, 0.78–0.97	0.69 ± 0.91, 0, 0–3, 0.59–0.78	7.93	*0.005*
	Acceptance		1.81 ± 0.63, 2, 0–3, 1.75–1.87	1.86 ± 0.68, 2, 0–3, 1.79–1.93	1.31	0.25
	Sense of humour		1.23 ± 0.80, 1, 0–3, 1.16–1.31	1.32 ± 0.78, 2, 0–3, 1.24–1.40	2.05	0.15

Statistically significant: *p* < 0.05; *p* < 0.01; *p* < 0.001.

Explanation: *n*, subgroup size; M, arithmetic mean; SD, standard deviation; Me, median; Min., minimum; Max., maximum; 95% CI, confidence interval for the mean; SES, global self-assessment score; PSS-10, stress intensity.

### 3.3 Correlation between global self-esteem, stress intensity and coping with stress strategies in the subgroups under study

**Figure 3:**
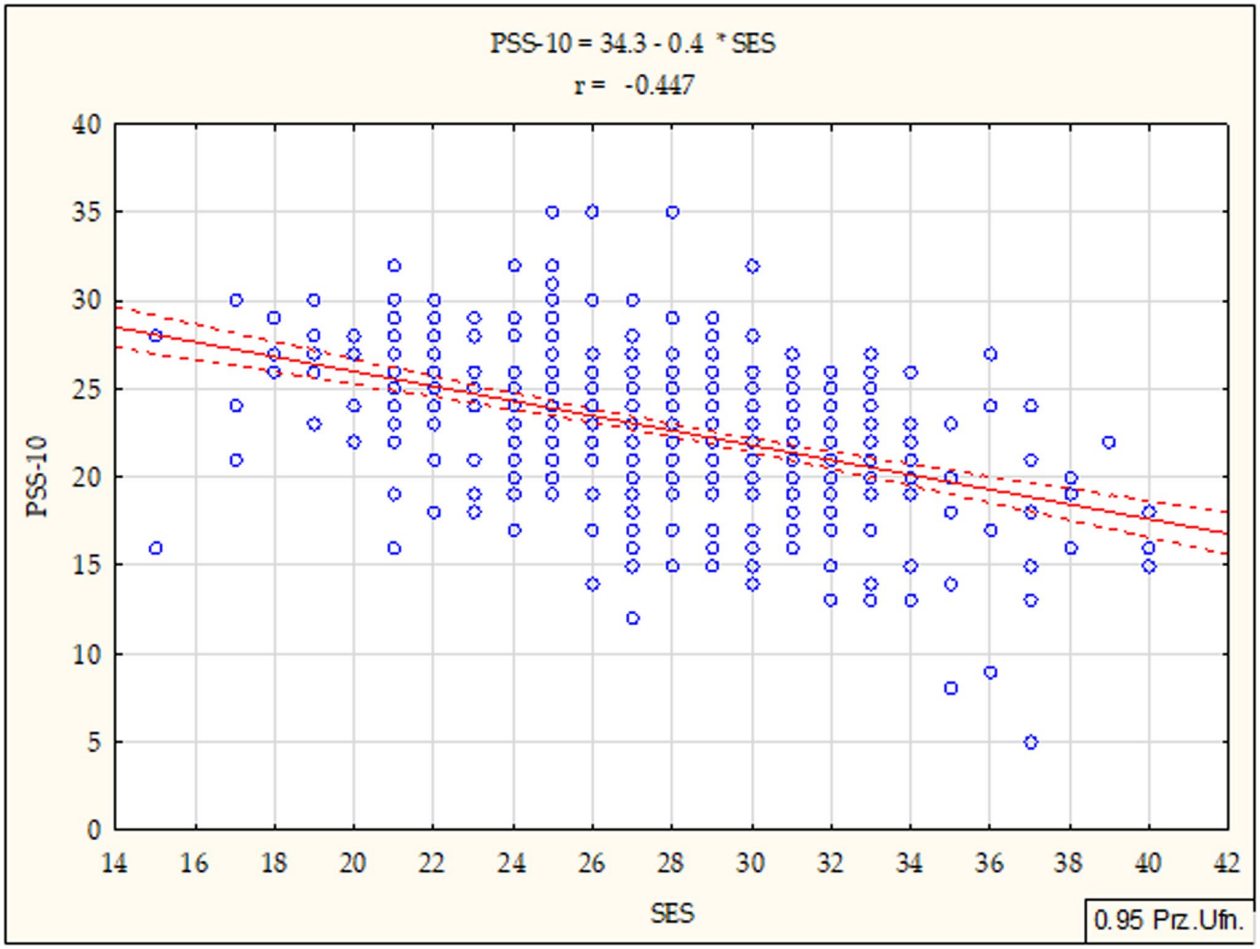
Diagram of dispersion showing a relationship between global self-esteem and stress intensity for subgroup 1. Statistically significant: *p* < 0.05; *p* < 0.01; *p* < 0.001. SES - Global self-esteem, PSS-10- stress intensity.

**Figure 4:**
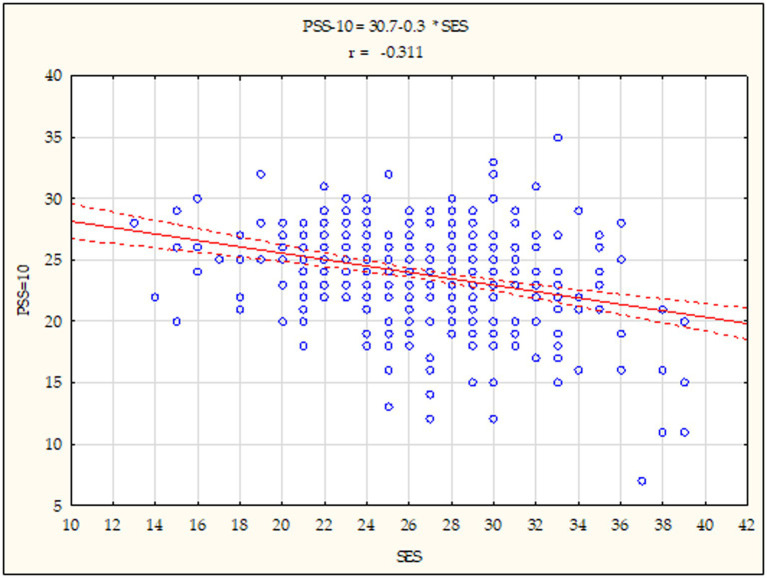
Diagram of dispersion showing a relationship between global self-esteem and stress intensity for subgroup 2. Statistically significant: *p* < 0.05; *p* < 0.01; *p* < 0.001. SES - Global self-esteem, PSS-10- stress intensity.

Further analyses involved the calculation of Pearson’s correlation coefficients (r) between the global self-esteem and the general index of perceived stress and the coping strategies among the students under study, determining the correlation strength and direction. The data presented in Figures 3, 4 indicates that both in subgroup 1 (*r* = −0.447; *p* < 0.001) and subgroup 2 (*r* = −0.311; *p* < 0.001), the analysed relationships were statistically significant, negative and with an average strength. Therefore, the results showed that students with a higher global self-esteem exhibit a significantly lower stress intensity and vice versa.

The correlation coefficients shown in Table 3 suggest statistically significant relations between global self-esteem and multiple coping strategies in the students under study, both in subgroup 1 and subgroup 2.

**Table 3:** Significance of the relationship between global self-esteem and coping with stress strategies in the subgroups under study.

No	Variables		SES			
			Subgroup 1		Subgroup 2	
(Mini-Cope)			r	*p*	r	*p*
1	Active coping	Active coping	0.255	*0.0001*	0.276	*0.0001*
		Planning	0.223	*0.0001*	0.256	*0.0001*
		Positive revalidation	0.285	*0.0001*	0.328	*0.0001*
2	Helplessness	Use of psychoactive substances	−0.182	*0.0001*	−0.0.202	*0.0001*
		Cessation of actions	−0.267	*0.0001*	−0.352	*0.0001*
		Self-blaming	−0.483	*0.001*	−0.532	*0.001*
3	Seeking support	Seeking emotional support	0.140	*0.004*	0.175	*0.01*
		Seeking instrumental support	0.088	0.07	0.128	*0.01*
4	Avoidance behaviours	Taking care of something else	−0.008	0.87	0.051	0.33
		Denial	−0.218	*0.0001*	−0.187	*0.0001*
		Discharge	−0.176	*0.0001*	−0.129	*0.01*
5	Turning to religion		−0.063	0.19	0.122	*0.02*
6	Acceptance		0.168	*0.0001*	0.211	*0.0001*
7	Sense of humour		0.063	0.19	−0.014	0.79

Statistically significant: *p* < 0.05; *p* < 0.01; *p* < 0.001.

There were positive and weak correlations between global self-esteem and active coping with stress strategies in subgroup 1, i.e., active coping (*r* = 0.255; *p* < 0.0001), planning (*r* = 0.223; *p* < 0.0001), positive revalidation (*r* = 0.285; *p* < 0.0001). Further analyses revealed significant, negative links between global self-esteem and avoidance strategies, leading to helplessness. There are noteworthy links with the self-blaming strategy. It is a negative correlation with the highest strength (on an average level) of all the correlations under analysis (*r* = −0.483; *p* < 0.001) in subgroup 1.

There were also positive and weak relationships in subgroup 2 between global self-esteem and active coping with stress strategies, i.e., active coping (*r* = 0.276; *p* < 0.0001), planning (*r* = 0.256; *p* < 0.0001) and, on an average level, positive revalidation (*r* = 0.328; *p* < 0.0001). The correlation coefficients between the global self-esteem and helplessness-related strategies had a negative direction and a strong negative correlation with the self-blaming (*r* = −0.532; *p* < 0.001) and an average strength correlation with the cessation of actions strategy (*r* = −0.352; *p* < 0.001) proved to be the most significant. Detailed data are provided in Table 3.

### 3.4 Predictors for stress intensity

Further analyses involved an attempt to determine predictors for stress intensity in the students under study in subgroups 1 and 2. In developing a model of multiple regression, stress intensity was adopted as a dependent variable, while the coping strategies and global self-esteem were taken as the pool of independent variables. The regression analysis showed three independent variables to be predictors for stress intensity among students in subgroup 1, explaining 26% of the score variability altogether (Table 4). The greatest contribution was assigned to the global self-esteem (20%). The regression coefficient was negative (*ßeta* = −0.314; *R*^2^ = 0.20), which is indicative of a negative correlation. This means that global self-esteem as a personal resource helps to handle the internal and external requirements regarded as a burden for students in studying during the COVID-19 pandemic.

**Table 4:** Summary of regression—predictors for the stress intensity among students during the COVID-19 pandemic in subgroup 1.

No	Variables	*R* ^2^	*ßeta*	*ß*	Error *ß*	*t*	*p*-value
1	SES	0.20	−0.314	−0.293	0.046	−6.393	0.000
2	Self-blaming	0.05	0.219	1.068	0.245	4.362	0.000
3	Doing something else	0.01	0.105	0.643	0.265	2.426	0.016
		*R* = 0.52; *R*^2^ = 0.27; adjusted *R*^2^ = 0.26					

Statistically significant: *p* < 0.05; *p* < 0.01; *p* < 0.001.

R, correlation coefficient; R^2^, multiple determination coefficient; ßeta, Standardised regression coefficient; B, non-standardised regression coefficient; Error B, non-standardised regression coefficient error; t, t test value.

When seeking predictors for the stress intensity among students during the COVID-19 pandemic in subgroup 2, it was demonstrated that the three variables explain 17% of the score variability (Table 5). The coping strategy characterised by self-criticism and self-blaming for the situation over the past month was the main predictor for the stress intensity. The regression coefficient was positive (*ßeta* = 0.279; *R*^2^ = 0.15), which is indicative of a positive correlation. It is clear that the self-blaming strategy can make the rational assessment of the situation difficult for the students. The other two variables (global self-esteem and active coping) are not significant in predicting stress intensity, as they explain merely 2% of the score variability.

**Table 5:** Summary of regression—predictors for the stress intensity among students during the COVID-19 pandemic in subgroup 2.

No	Variables	*R* ^2^	*ßeta*	*ß*	Error *ß*	*t*	*p*-value
1	Self-blaming	0.15	0.279	1.305	0.266	4.899	0.000
2	SES	0.01	−0.170	−0.143	0.050	−2.856	0.005
3	Active coping	0.01	0.150	0.840	0.307	2.738	0.006
		*R* = 0.41 *R*^2^ = 0.17.; adjusted *R*^2^ = 0.16					

Statistically significant: *p* < 0.05; *p* < 0.01; *p* < 0.001.

R, correlation coefficient; R^2^, multiple determination coefficient; ßeta, Standardised regression coefficient; B, non-standardised regression coefficient; Error B, non-standardised regression coefficient error; t, t test value.

A summary of the regression results shows that the main role in the prediction of the stress intensity among students in subgroup 1 is played by global self-esteem as a personal resource. The situation in subgroup 2 is different. The main determinant of the stress intensity among students in subgroup 2 is the coping strategy of the group indicative of helplessness, referred to as self-blaming.

## 4 Discussion

A literature review shows that students’ lives can be a significant cause of stress, as students report a higher level of stress than their non-student peers (42). Numerous studies confirm that students of healthcare schools can experience more stress than students of other schools as they are more exposed to academic, social and financial stressors (43). In particular, students of medical majors can encounter considerable stress in the clinical study environment, which is mainly associated with a lack of professional knowledge and skills, which can result in the choice of various coping strategies to alleviate such stress (44, 45).

### 4.1 Factors which affect self-esteem

The scores obtained by over half of the students were indicative of low global self-esteem. Moreover, 1/3 of the respondents had average self-esteem, whereas high self-esteem was determined in the others. These findings are similar to the reports of other authors.

Bodys-Cupak et al. (46) report that over half of nursing students had a strong sense of self-efficacy. The study conducted by Shrestha et al. (16) demonstrated that 95.3% of the nursing undergraduate students had a high level of self-esteem. High self-esteem levels were also observed in nursing students by Lopes Chaves (15). Altaweel et al. (47), Almansour (48), Banappagoudar (18), Ibrahim (17), and Mane (37) reported that a majority of nursing students had self-esteem at a moderate level. According to many scientists, this is the optimum level of self-esteem, as it has a positive impact on their health and helps to maintain constant learning and self-improvement progress. Moreover, many studies show that university nursing educators must develop effective strategies to enhance self-esteem among their nursing students (18). University administrators must also take the initiative and responsibility to create stress reduction courses and self-esteem building programs (48). A literature review shows that self-esteem is shaped by various interrelated factors, such as family, teachers, appearance, success, environment, experiences, university syllabuses, religion, relationships, social contacts (24, 49, 50).

The study showed that students with a higher global self-esteem exhibited a significantly lower stress intensity and vice versa. Moreover, a statistically significant relationship between global self-esteem and many coping strategies was observed among the students under study. A positive correlation was observed among medicine students between global self-esteem and active coping strategies, i.e., active coping, planning, positive revalidation, seeking emotional support, and acceptance. A positive correlation was demonstrated among veterinary medicine students between global self-esteem and active coping strategies, i.e., active coping and planning, at an average level, with the positive revalidation strategy.

A study conducted by Bodys-Cupak et al. (46) also showed that self-efficacy had a significant impact on stress levels and strategies for coping with difficult situations in nursing students. Individuals with a higher self-efficacy felt a low level of stress, and they applied the following coping strategies more often: active coping, planning, positive revalidation, acceptance and seeking emotional support. A study conducted by Molero (51) demonstrated a significant relationship between general self-efficacy and perception of stress. This means that individuals who perceived themselves as more efficacious experienced less stress arising from fear or anxiety, probably because they thought that they would be able to cope with the threatening situations. These findings are consistent with other studies that emphasise that perceived stress increases with decreased control of the situation and with decreased feelings of self-efficacy (46, 52, 53). Zhao et al. (54) also observed the importance of strengthening the feeling of self-efficacy in order to alleviate stress and build active coping strategies. Liu et al. (55) demonstrated that negative styles of coping were correlated positively with the general level of stress among nursing students, whereas positive coping styles were not correlated with the level of stress. Zhao et al. (54) and Hwang et al. (56) demonstrated in their studies that a higher level of stress associated with the clinical setting increased the probability that nursing students would choose avoidance strategies.

### 4.2 Level of stress

It was demonstrated in this study that a large majority of the participants felt a high level of stress regardless of the study major. A literature review shows that medical students experience a high level of stress due to their academic burden, lack of time for rest, and large amounts of material to study (57). Medicine students experienced particularly high levels of stress during the COVID-19 pandemic, as the pandemic had an impact on their physical, mental and emotional health.

According to some studies, medical students reported high levels of stress before the COVID-19 pandemic (56–60). The intensity of stress experienced by students varied considerably, depending on the syllabus and the grading system. Earlier studies at medical universities in various countries demonstrated various levels of stress experienced by students (61–63).

According to studies conducted in Germany, the USA and the UK, veterinary students also experience huge mental burdens, manifesting as a higher level of mental problems compared with the general population (64) and students of other majors (65). A study conducted by Platt et al. (65) confirmed that veterinary students experience a large mental burden, especially with higher levels of depression and anxiety than in the general population. An increased level of stress among students is a cause for concern as it has an impact on student mental health, thereby limiting their ability to use their greatest potential. Oura et al. (60) showed that nearly 50% of the respondents experienced stress at a considerable level, regardless of age, gender or faculty. Shaikh et al. (66) also showed that over 90% of the students experienced a variety of stress during their classes, and Garg et al. (58) confirmed that the intensity of stress experienced by medical students is dynamic and the causes of stress vary depending on the stage of the syllabus.

This study has shown that students in subgroup 2 used the coping strategies, i.e., active coping and planning, with greater intensity than students in subgroup 1, while students in subgroup 1 chose the strategy of positive revalidation significantly more often than the others. In a group of less effective strategies, referred to as helplessness, students in subgroup 1 obtained significantly higher scores for the cessation of actions strategy compared with students in subgroup 2. In stressful situations, students in subgroup 2 used a destructive strategy referred to as self-blaming more often than students in subgroup 1.

A study conducted by Babicka-Wirkus et al. (67) during the COVID-19 pandemic showed that Polish students chose mainly such coping strategies as acceptance, planning and seeking emotional support. On the other hand, using psychoactive substances, denial or withdrawal from behaviours and coping with the use of religion were the least often employed coping strategies during the pandemic.

According to the findings of a study conducted by Baluwo et al. (68), active coping and planning were the typical coping strategies among nursing students. Other studies confirm that students often choose active coping strategies (69, 70). A study conducted in the UK showed medical students abuse alcohol as a strategy for coping with stress (71). According to other studies, some people try to cope with difficult situations by smoking tobacco or using drugs (72). Unfortunately, many scientists confirm that many students experiencing a high level of stress abuse psychoactive substances (68, 73).

## 5 Limitations

This study has shown a correlation between self-esteem, the level of stress and the choice of a coping strategy. It has some noteworthy limitations. First, data were collected only at the university, so it is difficult to extrapolate the conclusions to other nursing students. Therefore, further research on nursing students’ self-esteem is needed. Second, the questionnaire used for data collection was completed by students themselves, which may lead to bias in reporting due to the way in which potential users perceive the questions. Furthermore, this is a descriptive and cross-sectional survey, focusing solely on the initial period of nursing students’ clinical practice. In future, we are going to continue studying predictors for stress and coping by nursing students during clinical practice in a longitudinal, multi-centre study.

## 6 Implication for practice

This piece of research can be used to develop educational programmes to teach students how to improve their self-esteem and decrease their level of perceived stress. Understanding factors that increase awareness has a significant impact on other aspects of students’ lives, including their physical and mental health, as well as a better understanding of their own self-worth. Therefore, an increase in the level of self-esteem can prevent emotional and behavioural problems and increase the level of motivation for studying, further self-improvement and development. Moreover, providing students with important guidelines concerning increasing their self-esteem can have a positive impact on their way of thinking and the quality of care provided by them. It is crucial to engage academic teachers and clinical mentors in enhancing students’ self-esteem through effective educational behaviour, proper competency development, and presentation of efficient coping strategies. This is especially important for novice nurses, midwives, and paramedics who encounter intense emotional experiences in their syllabuses and need to learn to cope with them. A valuable recommendation is also to provide students with access to immediate psychological assistance or peer and family support if needed. As part of the support, psychological interventions can be offered to students, such as psychoeducation, relaxation, planning daily schedules, and maintaining relationships (74, 75). It is also important to study the relationship between self-esteem and other associated factors among students of various majors.

## 7 Conclusion

The level of self-esteem was low in most of the students taking part in the survey. This can have an impact on their mental and physical health. Therefore, it is necessary to develop effective strategies aimed at increasing students’ self-esteem. Self-esteem had a significant impact on the stress level and methods of coping with difficult situations in medical students. According to this study’s findings, students experience a high level of stress regardless of their major. A majority of the students applied constructive strategies for coping with stress.

## Data Availability

The raw data supporting the conclusions of this article will be made available by the authors, without undue reservation.
